# Intrinsic immune evasion patterns predict temozolomide sensitivity and immunotherapy response in lower-grade gliomas

**DOI:** 10.1186/s12885-022-09984-5

**Published:** 2022-09-12

**Authors:** Zewei Tu, Qiankun Ji, Qing Han, Xiaoyan Long, Jingying Li, Lei Wu, Kai Huang, Xingen Zhu

**Affiliations:** 1 Department of Neurosurgery, The Second Affiliated Hospital of Nanchang University, Nanchang, Jiangxi 330006 P.R. China; 2 Institute of Neuroscience of Nanchang University, Nanchang, Jiangxi 330006 P.R. China; 3 Jiangxi Key Laboratory of Neurological Tumors and Cerebrovascular Diseases, Nanchang, Jiangxi 330006 P.R. China; 4 The First Clinical Medical College of Nanchang University, Nanchang, P.R. China; 5 East China Institute of Digital Medical Engineering, Shangrao, China; 6 Department of Comprehensive Intensive Care Unit, The Second Affiliated Hospital of Nanchang University, Nanchang, P.R. China

**Keywords:** Lower-grade glioma (LGG), Prognosis, Cancer immune evasion, Immunotherapy response, Temozolomide (TMZ) sensitivity

## Abstract

**Background:**

Although intrinsic immune-evasion is important in cancer proliferation, metastasis and response to treatment, it is unclear whether intrinsic immune-evasion patterns of gliomas can aid in predicting clinical prognosis and determining treatment.

**Methods:**

A total of 182 immune-evasion genes intrinsic to cancer were subjected to consensus clustering to identify immune-evasion patterns in 1421 patients with lower-grade glioma (LGG). The levels of each cancer hallmark were determined by the Gene Set Variant Analysis (GSVA) method, and immune cell infiltrations were quantified using two algorithms, the single-sample Gene Set Enrichment Analysis (ssGSEA) and the Cell-type Identification By Estimating Relative Subsets Of RNA Transcripts (CIBERSORT) methods. IEVscore was determined by a method that combined univariate Cox regression analysis, least absolute shrinkage and selection operator (LASSO) regression and principal component analysis (PCA).

**Results:**

Transcriptional and genomic analysis showed that most immune evasion genes (IEVGs) were upregulated in LGGs, with aberrant expression driven by alterations in copy number variants (CNV). Based on the mRNA expression profiles of cancer-intrinsic IEVGs could be divided into three LGG subgroups with distinct prognosis, clinicopathological features and immune infiltrations. A combined scoring scheme designed to assess the immune-evasion levels of LGGs divided these 1421 patients into two subgroups that differed in IEVscores. LGG patients with low-IEVscore had a better prognosis, would be more likely to benefit from immune check-point inhibitors and would be more susceptible to temozolomide (TMZ) chemotherapy.

**Conclusion:**

Intrinsic immune evasion in the tumor microenvironment (TME) has a crucial effect on glioma formation. Quantitatively assessing the IEV scores of individual LGG patients could enhance knowledge about the intra-glioma microenvironment and lead to the development of individualized therapeutic strategies for patients with LGG.

**Supplementary Information:**

The online version contains supplementary material available at 10.1186/s12885-022-09984-5.

## Background

Phenotypic alterations of oncocytes allow these cells to escape recognition and hunting by effector-cells of the host-immune system, including CD8 + T cells, macrophages and nature killer (NK) cells [1]. These phenotypic alterations not only affect tumorigenesis and tumor progression but also determine the therapeutic effects of immunotherapeutic agents, including immune check-point blockers (ICB) and chimeric-antigen receptor T (CAR-T) cells. Genomic research to date has identified several mechanisms, including loss of antigen presentation machinery (APM) and defects in interferon signaling pathway, that could enhance the ability of cancer cells to escape the immune system and to avoid being killed by immune effector cells [2–4]. The vital importance of immune evasion in onco-immunology and the need to formulate cancer immunotherapy strategies precisely and individually suggest the need to focus on the mechanisms underlying immune evasion and methods of avoiding immune evasion [5–7].

Malignant diffuse glioma has the highest fatality rate among patients with intracranial tumors, is characterized by diffuse infiltration, high recurrence and reduced sensitivity to drugs [8, 9]. The World Health Organization (WHO) has classified gliomas into grades of I, II, III and IV, and WHO I and II gliomas are defined as low-grade glioma (LGGs) and WHO III and IV are high-grade gliomas (HGGs). Considering the satisfactory prognosis of WHO grade I glioma, the Cancer Genome Atlas (TCGA) classified WHO grades II and III gliomas as lower-grade gliomas (LGGs) and WHO grade IV gliomas (glioblastoma, GBM) as a separate category. About 29% of central nervous system (CNS) tumors are LGGs, with these patients having a median overall survival time of approximately 7 years, compared with 5 years in patients with GBM [10]. Generally, the standard therapy for patients with LGG consists of surgical resection followed by temozolomide (TMZ) chemotherapy and/or radiotherapy. Because these tumors have a high recurrence rate, are generally resistant to chemotherapy, and can easily progress to GBM, conventional therapy becomes less effective, indicating a need for new therapeutic strategies.

Tumor immunotherapy strategies have been found to benefit tumor patients, including those with glioma [6]. Because the blood–brain barrier (BBB) blocks most drugs in the peripheral blood vessels from passing into the CNS, and because the brain is a natural immunosuppressive microenvironment, little is known about the efficacy and safety of glioma immunotherapy [11, 12]. Improving understanding of immune evasion patterns and mechanisms, as well as the underlying biological processes and pathways of the glioma microenvironment is essential for improving treatment responses. Heterogeneity within and between gliomas may assist in the design of individual therapeutic strategies precisely targeted to the novel molecular characteristics of individual LGG patients. Although knowledge of intrinsic immune-evasion mechanisms remains incomplete, cancer cell evasion of the immune system plays a crucial role in enabling these cells to avoid being killed by immunologic effector cells. Thus, blocking immune evasion can enhance the efficacy of cancer therapy and benefit cancer patients.

The present study investigated the implications of intrinsic immune-evasion genes in the immune microenvironment, as well as their clinicopathological relevance and association with the prognosis of patients with LGGs by integrating the genetic and transcriptomic data of 1421 LGG patients from public datasets. Unsupervised consensus cluster analysis of 88 prognostic cancer-intrinsic immune-evasion genes resulted in the identification of three LGG clusters with distinct immune-infiltration status and clinical outcomes. An individual scoring system was formulated to determine the cancer immune evasion status, clinical prognosis, immunotherapy response, and TMZ sensitivity of LGGs. This immune evasion associated scoring system could provide benefit for LGG patients in determining postoperative therapeutic strategies for clinical application.

## Methods and materials

### LGG cohorts collection and preprocessing

Data were collected from six independent LGG cohorts, including transcriptional expression on immune evasion genes (IEVGs) and corresponding survival information of 1421 LGG patients. Cohorts analyzed included the TCGA-LGG (*n* = 457), GSE16011 (*n* = 103), GSE61374 (*n* = 137), GSE108474 (*n* = 141), CGGA_mRNA_325(*n* = 172) and CGGA-mRNA_seq693 (*n* = 379) cohorts. Patients were included if they had undergone surgery and had a postoperative overall survival (OS) time > 30 days. RNA expression data of the TCGA-LGG cohort were downloaded from the UCSC Xena datasets (https://xenabrowser.net/), with the format of fragments per kilobase per million (FPKM) converted into transcripts per kilobase per million (TPM). Associated clinicopathological information was obtained from the cBioPortal database (https://www.cbioportal.org/). RNA-seq profiles and relative clinical matrices of the CGGA cohorts were downloaded from the Chinese Glioma Genome Atlas (CGGA) database (http://www.cgga.org.cn/download.jsp). Raw data were acquired for the three cohorts in the Gene Expression Omnibus (GEO) repository (https://www.ncbi.nlm.nih.gov/gds), with the data adjusted by the robust-multiarray averaging (RMA) normalization method using the “affy” [13] and “simpleaffy” [14] packages in the R programming environment. Corresponding clinicopathological data of the three GEO microarray cohorts were obtained from previous publications. The “combat” function in the “sva” [15] R package was utilized to remove the batch-effects between the two CGGA cohorts or among the three GEO cohorts, with the merged LGG cohorts called the meta-GEO and meta-CGGA cohorts, respectively. In addition, the “maf” file of single-nucleotide polymorphisms (SNPs) and the copy number variation (CNV) matrix of the TCGA-LGG cohort were acquired from the UCSC Xena website. The clinical and pathological information on these LGGs is described in Table 1. The source of immune-evasion genes were obtained from the previous research [1], and the gene list were shown in Supplementary Table 1.

**Table 1:** Summary of clinical characteristics of patients with LGG in six datasets

Characteristics	TCGA dataset	CGGA_325 dataset	CGGA_693 dataset	GSE16011 dataset	GSE61374 dataset	Rembrandt dataset
**No. of patients**	457	172	379	103	137	141
**Platform**	Illumina RNAseq	Illumina HiSeq	Illumina HiSeq	Affymetrix U133 Plus 2.0 Array	Affymetrix U133 Plus 2.0 Array	Affymetrix U133 Plus 2.0 Array
**Age (years)**						
Range	14–87	10–74	11–69	23–81	21–80	17–87
Median	41	39	40	44	41	42
**Gender**						
Female	201	66	167	36	53	47
Male	256	106	212	67	84	72
Unknown	0	0	0	0	0	22
**WHO grade**						
II	216	98	153	22	61	76
III	241	74	226	81	76	65
**IDH mutation status**						
Yes	369	125	262	45	115	
No	86	44	80	37	22	
Unknown	2	1	37	21	0	
**1p/19q codeletion status**						
Yes	151	55	122	37	37	
No	306	115	255	39	100	
Unknown	0	2	2	27	0	
**Oveall survival (year)**						
Range	0.01–17.34	0.06–13.18	0.14–13.78	0.19–20.68	0–17.7	0.08–20.69
Median	1.22	6.05	3.98	3.3	4.4	3.16

### Collection of clinical samples and western blotting

Seven pairs of LGG and adjacent tissue samples were collected postoperatively from patients with LGG who underwent surgery in the Neurosurgery Department of The Second Affiliated Hospital of Nanchang University (NCUSAH) in 2021. All patients provided written informed consent for intraoperative collection of tissue samples. Tissues were excised during surgery and stored in liquid nitrogen. The acquisition and use of these clinical samples were in strict accordance with the guidelines of the Medical Ethics Committee of NCUSAH.

The reagents and procedures for protein extraction and western blotting have been described [16]. Rabbit polyclonal antibodies against CEP55 (catalog number 23891–1-AP, diluted 1:500) and GAPDH (catalog number 10494–1-AP, diluted 1:5000) were purchased from the Proteintech company. Horseradish peroxidase (HRP)-conjugated affinipure goat anti-rabbit IgG (catalog number SA00001-2, diluted 1:2000) was also purchased from Proteintech company.

### Unsupervised consensus cluster analysis and metascape enrichment analysis

Based on the mRNA expression profiles of the IEVGs or IESig genes, consensus clustering was performed using the “ConsensusClusterPlus” R package [17]. The Euclidean-distance was used to calculate the similarity distance, and the k-means cluster method was utilized to perform clustering according to 100 iterations, with 80% of patients included in each iteration. The gene enrichment analysis was performed in the webtool of a wide-used bioinformatic website “Metascape” (http://metascape.org/) [18]. Biological processes of gene ontology and KEGG pathway datasets [19–21], obtained from the Molecular Signatures Database (MSigDB, https://www.gsea-msigdb.org/) [22, 23], were used to analyze the enrichment terms of IEVGs.

### Gene Set Variation Analysis (GSVA) and well-defined biological process signatures

Based on the “GSVA” algorithm [24], the “gmt” file of Hallmark gene sets retrieved from the Molecular Signatures Database (MSigDB, https://www.gsea-msigdb.org/gsea/msigdb/, version 7.4) was used to calculate the scores of the processes for the LGG samples. A sequence of well-designed signatures had been constructed to quantify relative classical and vital biologic processes, such as angiogenesis, immune check-point, antigen processing machinery (APM), CD8 T cell effector, epithelial-mesenchymal transition markers (including EMT1/2/3), WNT targets, DNA-damage repair, mismatch repair, nucleotide excision repair, FGFR related genes, pan-fibroblast TGFb response signature (Pan-F-TBRS), cell cycle, cell-cycle regulators, Fanconi anemia, homologous recombination, DNA replication and KEGG discovered histones [19]. The method described in the previous study [25] was applied in the present study to score the processes for each LGG sample.

### Single-sample Gene Set Enrichment Analysis (ssGSEA) and CIBERSORT

To assess the abundance of immune cells that had infiltrated the tumor microenvironment (TME), 28 elaborately defined immune cell signatures were collected as described [26], and the levels of infiltration of various immune cells in each LGG sample were quantified using a ssGSEA algorithm [27]. The abundance of 22 distinct immune cell types in LGG samples was also determined using the CIBERSORT algorithm [28], a type of deconvolution method, based on the specific transcriptional profiles of these LGG samples.

### Screening of Differentially Expressed Genes (DEGs) among different immune evasion subgroups

Distinctive DEGs in each IEV cluster were assessed using the “voom” function of the R-package “limma” [29], which normalized the mRNA expression profiles of LGGs to set RNA-seq data suitable for linear modelling. Subsequently, the statistical significance of DEGs between each pair of IEV clusters was calculated using the “eBayes” function of “limma”. The filtering criteria for DEGs were defined as an adjusted *p*-value < 0.05 and a |log2 (fold change) |> 1.

### Determination of IEVscores

The immune evasion level of individual LGG patients was quantified by a cancer-intrinsic immune-evasion scoring scheme based on principal component analysis (PCA). Univariate Cox regression analysis was performed to evaluate the overlapping DEGs that intersected from the three IEV clusters. DEGs that showed prognostic significance were identified for subsequent least absolute shrinkage and selection operator (LASSO) regression modelling using the R-package “glmnet” [30] using a tenfold cross-validation method. The mRNA expression profile of the finally selected screened genes was used to perform PCA analysis; the first two principal components 1 (PC1) and 2 (PC2) were obtained and used to construct the signature score. Consistent with previous studies [31, 32], PC1 and PC2 were used to determine the formula defining the IEVscore:

$$IEscore=\Sigma PC{1}_{\mathrm{i}}+\Sigma PC{2}_{\mathrm{i}}$$

where i is the relative expression of the final determined DEGs.

### Tumor Immune Dysfunction and Exclusion (TIDE) and ESTIMATE immune cell infiltration algorithm

The Tumor Immune Dysfunction and Exclusion (TIDE) algorithm, which could quantify the dysfunction level of cytotoxic T lymphocytes (CTLs) and the exclusion levels of CTLs by immune-suppressors and predict the immunotherapy responses of patients based on expression profiles [33], was utilized to evaluate mechanisms of tumor immunologic evasion. The Estimate of Stromal and Immune Cells in Malignant Tumor Tissues from Expression Data (ESTIMATE) algorithm utilizes transcriptional data to estimate tumor cellularity and purity [34]. Based on this algorithm, scores representing infiltrated immune, stromal and/or cancer cell abundances were evaluated. ESTIMATE scores were higher and tumor purity lower in LGG tissues with higher immune and stromal cell infiltration levels.

### Acquisition of immunotherapy cohorts

To validate the ability of IEVscore to predict immunotherapy responses, two cancer cohorts in which patients received ICI therapy were evaluated: the IMvigor210 cohort, in which patients were treated with the anti-PDL1 agent atezolizumab [35], and the Vanallen cohort, in which patients were treated with the anti-CTLA-4 agent ipilimumab [36].

### TMZ sensitivity analysis

Because temozolomide (TMZ) is the current first-line chemotherapeutic drug for glioma treatment, determining the association between TMZ sensitivity and IEVscore is also promising clinically. TMZ sensitivity information for 835 cancer cell lines (CCLs) was collected from the Cancer Therapeutics Response Portal (CTRP version.2.0, https://portals.broadinstitute.org/ctrp) database [37–39], and 482 CCLs were collected from the Profiling Relative Inhibition Simultaneously in Mixtures (PRISM) Repurposing (https://depmap.org/portal/prism/) database. The areas under the curve (AUC), which represent the dose–response values and were positively associated with drug-tolerance ability (higher AUC represents greater resistance to TMZ), were obtained for these two databases. Missing AUC values were replenished using the method of K-nearest neighbor (k-NN) imputation with the R-package “impute”. Because the drug sensitivity of the CCLs from the two databases had been obtained from the Cancer Cell Line Encyclopedia (CCLE) dataset, corresponding molecular data were acquired to simulate TMZ-sensitivity. Ridge regression, a method of model-tuning usually used to analyze multicollinear parameters, was applied to evaluate the TMZ sensitivity (as determined by AUC) for each LGG patient by performing analysis using the “pRRophetic” package [40].

### Statistical analysis

All statistical analyses were performed using the R programming environment (version 4.0.1). Normally distributed continuous data were quantitatively compared in two groups by Student’s t-test, whereas non-normally distributed data were compared by Wilcoxon rank-sum test [32]. The levels of expression of genes in paired LGG and adjacent tissue samples were compared by paired t-tests. Statistical comparisons among more than two groups were assessed by Kruskal–Wallis and one-way ANOVA methods for nonparametric and parametric tests, respectively. The prognosis of two LGG subgroups was determined by the Kaplan–Meier method and compared by log-rank tests, using the R-package “survminer” (0.4.9), with and the “surv-cutpoint” function of “survminer” was used to determine the most significant cut-off point to classify LGG patients [31]. The prognostic predictive power of IEVscore was evaluated by receiver operator characteristic (ROC) analysis using the “timeROC” (0.4) package. Statistical correlations between two variables were determined by Spearman correlation analysis. The prognostic role of IE-related genes was evaluated by univariate Cox regression analysis.

## Results

### The genetically altered landscape of immune-evasion regulators in LGGs

**Fig. 1:**
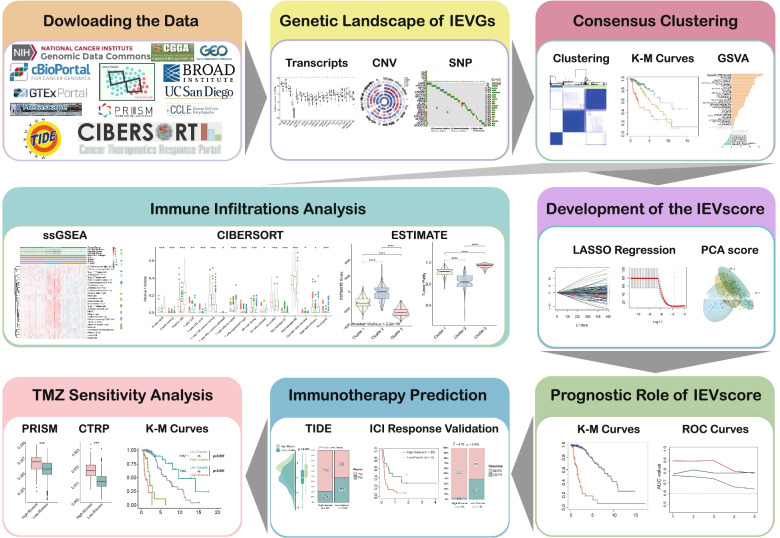
The research workflow of our study

**Fig. 2:**
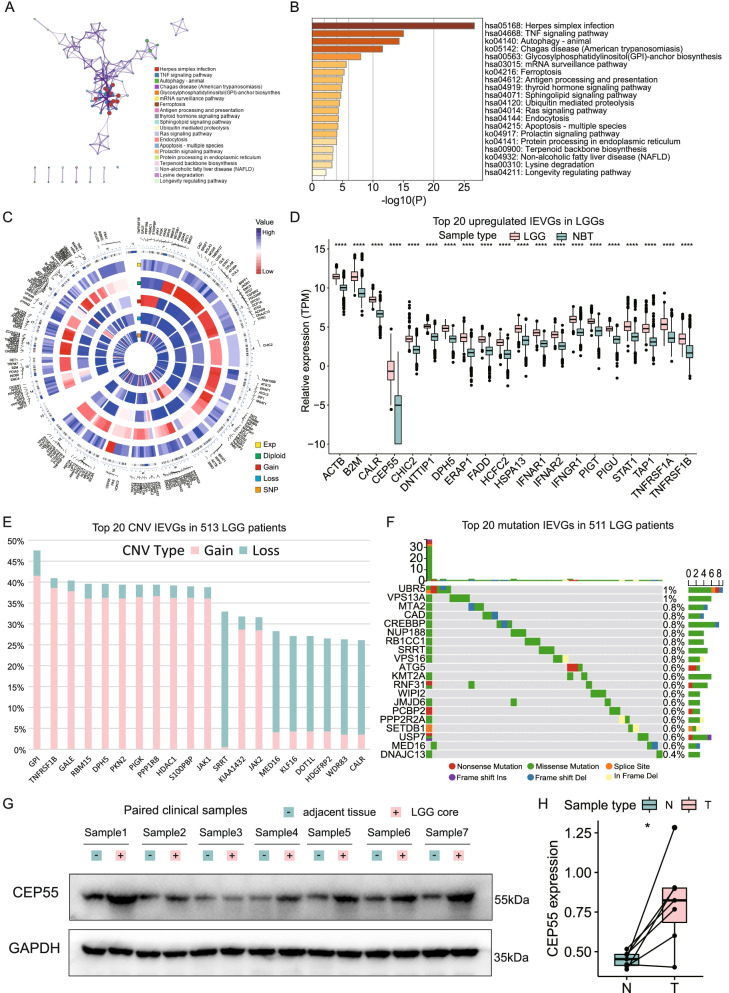
The multi-omics aberrant alterations of IEVGs in LGGs. **A** Biological process and pathway enrichments visualization of the 182 IEVGs showed the interactions among the enriched terms. **B** Biological process and pathway enrichments ordered by statistical significance. **C** Circus heatmaps represented the expression levels of transcriptomic data and genomic alteration proportions of IEVGs. **D** The boxplots showed the top 20 upregulated IEVGs in LGG patients. **E** The bar plots represented the top 20 frequent CNV IEVGs in LGGs. **F** The waterfall plot showed the SNP proportion of the top 20 mutation IEVGs in LGGs. The labelled asterisk indicated the statistical *p* value (****p < 0.0001)

The workflow of this study is depicted in Fig. 1. To assess the genomic alterations of IEVGs in LGGs, the transcriptional expression, copy number variations (CNVs) and single nucleotide polymorphisms (SNPs) of 179 cancer-intrinsic immune evasion genes were evaluated (3 of 182 IEVGs were unmapped in the TCGA cohort). Enrichment analysis of these 179 IEVGs using the Metascape webtool showed that, when GO-biological processes and KEGG pathway terms were included, these IEVGs were mainly enriched in the tumor necrosis factor (TNF) signaling pathway, autophagy, ferroptosis, antigen processing and presentation, ubiquitin mediated proteolysis, Ras signaling pathway, apoptosis, and protein processing in the endoplasmic reticulum (Fig. 2A, B). Several of these processes and pathways were previously shown to be essential in regulating cancer immune evasion [41–43]. The overall differences of transcriptional expression between LGGs and normal brain tissues (NBTs), including the CNV and SNP proportions of each IEVG in LGGs, were visualized using a circus heatmap (Fig. 2C). In this circus heatmap, the location of each IEVG is depicted on chromosomes, and the expression levels compared with NBTs, CNVs (including diploid, gain and loss) and SNP proportions are shown in the inner heatmaps. Blue indicates high expression or proportion, whereas red indicates lower expression or proportion. Most of the 179 IEVGs were upregulated in LGGs compared with NBTs, with the top 20 upregulated IEVGs shown in the boxplots (Fig. 2D). Ninety-three IEVGs were significantly overexpressed in LGGs, with the most upregulated IEVG being *CEP55*. The top 20 IEVGs with most frequent CNVs are shown in Fig. 2E. The alterations in CNV proportions of 14 IEVGs were > 30%, whereas the alterations of 130 IEVGs were > 10%. *GPI* was the IEVG with most frequent CNVs in LGGs (47.56%; gain 41.52%; loss, 6.04%). Analysis of mutation frequency of IEVGs in LGGs showed that all of the IEVGs had mutation frequencies < 1% (Fig. 2F), indicating that mutations of IEVGs had little effect on LGG immune evasion. Taken together, these findings show that aberrant genomic alterations were present in 172 IEVGs of LGGs, with CNV being the primary type of IEVG alterations. Because *CEP55* was the most significantly upregulated gene in LGGs, the expression of CEP55 protein was analyzed in seven paired LGG cores and adjacent tissues (Fig. 2G, full-length gels are presented in Figure S1), with these findings showing that the CEP55 protein was also upregulated in LGGs (Fig. 2H).

### Identification of intrinsic immune-evasion patterns in LGGs

**Fig. 3:**
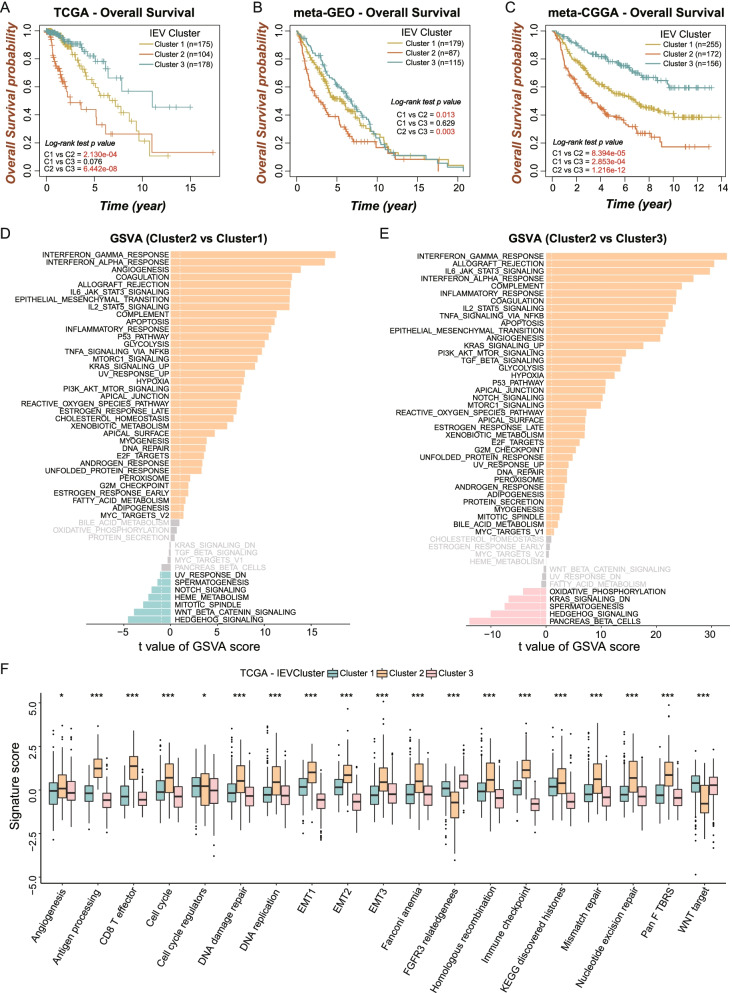
The clinical implications and relative biological processes of IE clusters in LGGs. **A-C** The survival curves showed the distinct prognosis among the three IE clusters in the TCGA **(A**, *n* = 457**)**, meta-GEO **(B**, *n* = 381**)** and meta-CGGA **(C**, *n* = 583**)** LGG cohorts. **D** Bar plot represented the significantly enriched terms of hallmark between Cluster2 (*n* = 104) and Cluster1 (*n* = 175) by GSVA analysis. **E** Bar plot represented the significantly enriched terms of hallmark between Cluster2 (*n* = 104) and Cluster3 (*n* = 178) by GSVA analysis. **F** The boxplots showed the distinct levels of 19 well-designed biological processes enrichment among the three clusters. The labelled asterisk indicated the statistical *p* value (*p < 0.05, ***p < 0.001)

The cancer-intrinsic immune-evasion patterns in the LGG TME were assessed by unsupervised clustering of LGG patients based on the transcriptional profile of 88 prognostic IEVGs that had been identified by univariate Cox regression analysis. Clustering analysis showed that k = 3 was the optimal cluster number in LGG samples (Figure S2A-D). Clustering analysis divided LGG patients in the TCGA-LGG cohort into three distinct clusters, with Clusters 1, 2, and 3 consisting of 175, 104, and 178 patients, respectively. Unsupervised clustering of the two merged LGG cohorts, using k = 3 as the optimal clustering number k = 3 was also performed. In order to investigate the potential clinical implications of the IEVG-based cluster, overall survival (OS) was compared in each pair of clusters in each LGG cohort. In the TCGA cohort, LGG patients in Cluster 2 had the poorest clinical prognosis when compared with patients in Clusters 1 (*p* = 0.0002) and 3 (*p* < 0.0001), whereas the difference between Clusters 1 and 3 was not statistically significant (*p* = 0.076) (Fig. 3A). Kaplan–Meier survival analysis in the meta-GEO yielded consistent results, indicating that OS was poorest in Cluster 2 (Fig. 3B). Further analysis of the meta-CGGA cohort showed that patients in Cluster 3 had significantly better prognosis than patients in Clusters 1 (*p* = 0.00028) and 2 (*p* < 0.0001), with the difference between Clusters 1 and 2 also being significant (*p* < 0.0001) (Fig. 3C). These findings indicated that the clusters might lead to promising clinical applications in patients with LGG.

To further analyze the underlying differences in biological processes and/or pathways between Cluster 2 and the other two clusters, GSVA scoring of hallmarks for LGG patients were compared in these clusters. Immune-activated associated processes, including interferon-γ, interferon-α, and inflammatory responses and TNFA signaling via NFKB, were significantly enriched in Cluster 2 compared with Clusters 1 and 3 (Fig. 3D, E). In addition, previously described signature scores were compared in the three IE clusters (Fig. 3F), with signature scores for antigen processing, CD8 T effector and immune checkpoint being much higher in Cluster 2 than in Clusters 1 and 3, similar to the GSVA results. Other characteristics of malignancy, including angiogenesis, cell cycle associated processes, EMT markers and DNA repair signatures, were also significantly higher in Cluster 2, whereas stromal associated signatures, including FGFR3 related genes and WNT target, were lowest in Cluster 2. These findings showed that the application of unsupervised clustering based on the profiles of IEVGs in LGGs could classify LGG into three subgroups, with distinct clinical prognosis and tumor immune phenotypic signatures. The abundance of immune-cell infiltration was subsequently incorporated to show infiltration differences among the three clusters.

### Associations between LGG cancer-intrinsic immune-evasion patterns and distinct immune cell infiltration

**Fig. 4:**
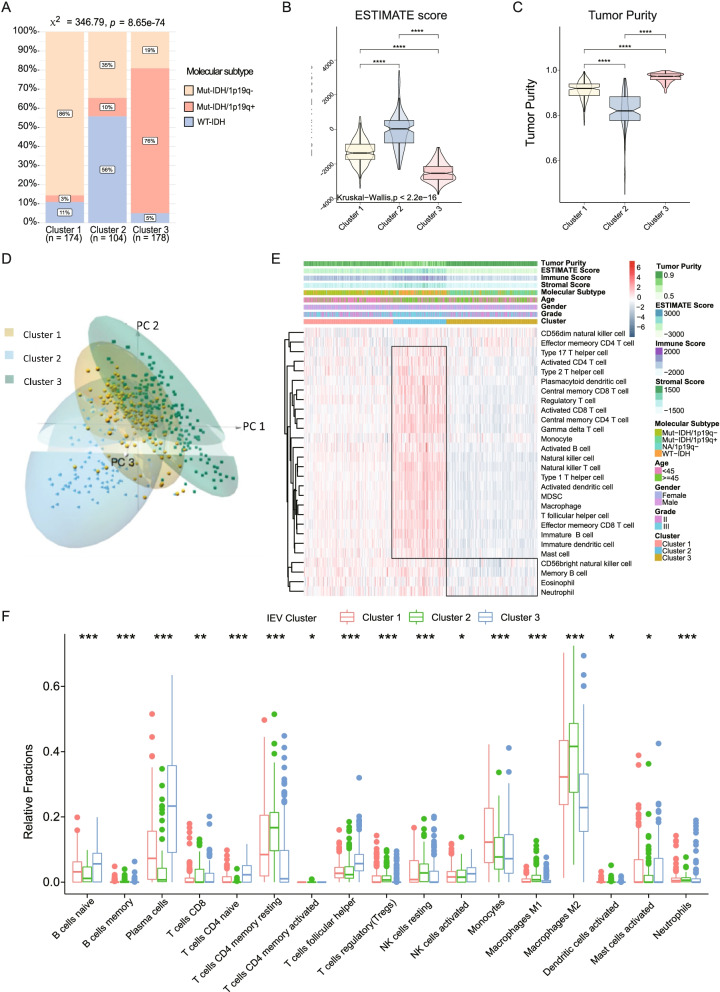
The distinct IE clusters showed different immune infiltrations levels. **A** The bar plots represented the proportions of different IDH mutation and 1p/19q co-deletion status among the three IE clusters. **B-C** The violin plots showed the distinct levels of ESTIMATE score and tumor purity among the three IE clusters. **D** Three-dimensional principal component analysis (PCA) indicated that the three IE clusters had distinct whole transcriptional expression profiles. **E** The heatmap showed the 28 types of immune cell infiltration levels among the three IE clusters by using ssGSEA algorithm. **F** The boxplots showed the 17 immune cell discrepancies among the three IE clusters by performing CIBERSORT deconvolution algorithm. The labelled asterisk indicated the statistical *p* value (*p < 0.05, **p < 0.01, ***p < 0.001, ****p < 0.0001)

To further evaluate the molecular characteristics and immune cell infiltration among the three clusters, *IDH* mutations and 1p/19q co-deletion status were compared. Significant differences were observed among the three clusters, with most LGGs in Cluster 1 containing *IDH* mutations and 1p/19q non-codeletions, most LGGs in Cluster 2 containing *IDH*-wild type, and most LGGs in Cluster 3 containing *IDH*-mutations and 1p/19q codeletions (Fig. 4A). LGGs in Cluster 2 had the highest ESTIMATE score, which correlated positively with immune cell infiltration level, and the lowest tumor purity, whereas LGGs in Cluster 3 had the lowest ESTIMATE score and the highest tumor purity (Fig. 4B, C). These findings indicated that LGGs in Cluster 2 were infiltrated by larger numbers of immune and/or stromal cells, whereas LGGs in Cluster 3 LGGs were not infiltrated by extraneous cells, with LGGs in Cluster 1 showing some immune cell infiltration.

Principal component analysis (PCA) showed that the three LGG Clusters occupied distinct spaces in the three-dimension PCA model based on whole transcriptional profiles (Fig. 4D), indicating that transcriptional discrepancies may lead to differences in immune-evasion among LGGs. Quantification of the abundance of 28 immune cell types in each LGG sample using the ssGSEA algorithm showed significant differences in immune cell infiltrations among the three LGG Clusters (Fig. 4E). CIBERSORT quantification of the levels of infiltration of various immune cells showed that CD8 + T cells, CD4 + memory resting T cells, regulatory T cells and M2 macrophages were enriched in the LGGs in Cluster 2, whereas follicular helper T cells were enriched in Cluster 3 (Fig. 4F).

### Identifying DEGs among the three LGG clusters

**Fig. 5:**
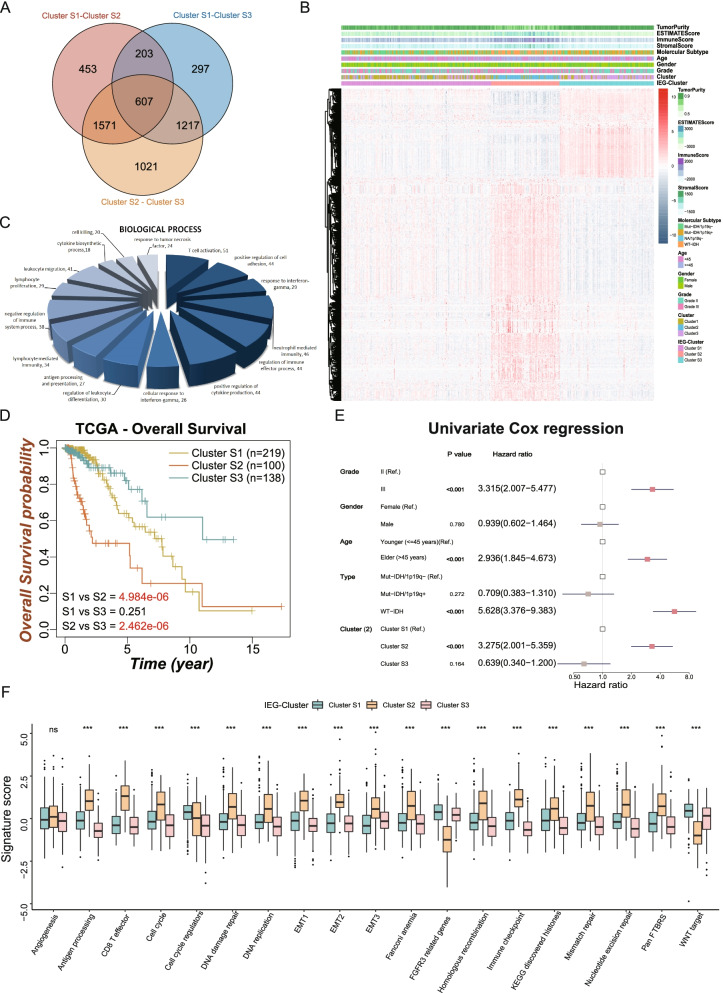
Identification of IEV signature genes across LGG patients. **A** The venn diagram represented the number of DEGs between each two clusters and 607 DEGs were identified as IEV signature genes. **B** The heatmap represented the levels of IEV signature genes. **C** Gene ontology biological processes analysis were performed to recognized the enriched terms of IEV signature genes. **D** Kaplan–Meier survival curves showed the distinct prognosis of Cluster S1 (*n* = 219), Cluster S2 (*n* = 100) and Cluster S3 (*n* = 138). **E** The forest plot showed the results of univariate Cox regression analysis and indicated that the Cluster S2 is a risk factor compared with other two Clusters. **F** The boxplots showed the distinct levels of 19 well-designed biological processes enrichment among the three IEG-Clusters.The labelled asterisk indicated the statistical *p* value (ns *p* > 0.05 and ***p < 0.001)

Although LGGs were clustered into three immune-evasion phenotypes, the potential differences in transcriptional alteration among these clusters were not clearly understood. The differences in transcriptional expression between each pair of clusters were investigated using a Bayesian method to recognize overlapping differentially expressed genes (DEGs). A total of 607 DEGs that represented crucial distinguishing scores of the three LGG clusters were considered IEV signature genes (Fig. 5A). Unsupervised consensus cluster analysis of the 607 DEGs in the TCGA-LGG cohort yielded three novel stable transcriptomic LGG clusters (Figure S3 A–D; k = 3), with a heatmap showing the expression patterns of the 607 DEGs in the three clusters (Fig. 5B). Gene ontology biological processes (GO-BP) analysis of the 607 DEGs showed enrichment of many onco-immunology related processes, including T cell activation, response to interferon-γ, neutrophil mediated immunity, and regulation of immune effector processes (Fig. 5C). These results further indicated that the 607 DEGs were associated with immune evasion processes and that the novel classifications might have clinical implications. Evaluation of survival showed that LGGs in Cluster S2 were associated poorer prognosis than the LGGs in the other two clusters (Fig. 5D). Moreover, univariate Cox regression analysis indicated that Cluster S2 was associated with a significant risk than Cluster S1 (HR = 3.28; 95% CI: 2.00–5.36, *p* < 0.001), whereas risks did not differ significantly in Clusters S3 and Cluster S1 (HR = 0.64; 95% CI: 0.34–1.20, *p* = 0.164) (Fig. 5E). Comparisons of the levels of 19 biological processes and pathway signatures showed that LGGs in Cluster S2 were significantly enriched in immune response processes, including antigen processing, CD8 T effector, immune checkpoint, EMT associated signatures (EMT1/2/3), cell cycle and DNA repair related signatures, including cell cycle, DNA damage repair, DNA replication, homologous recombination and mismatch repair, whereas FGFR3 related genes and WNT target signatures were enriched in Clusters 1 and 3.

### Determination of IEVscores and their clinical implications

**Fig. 6:**
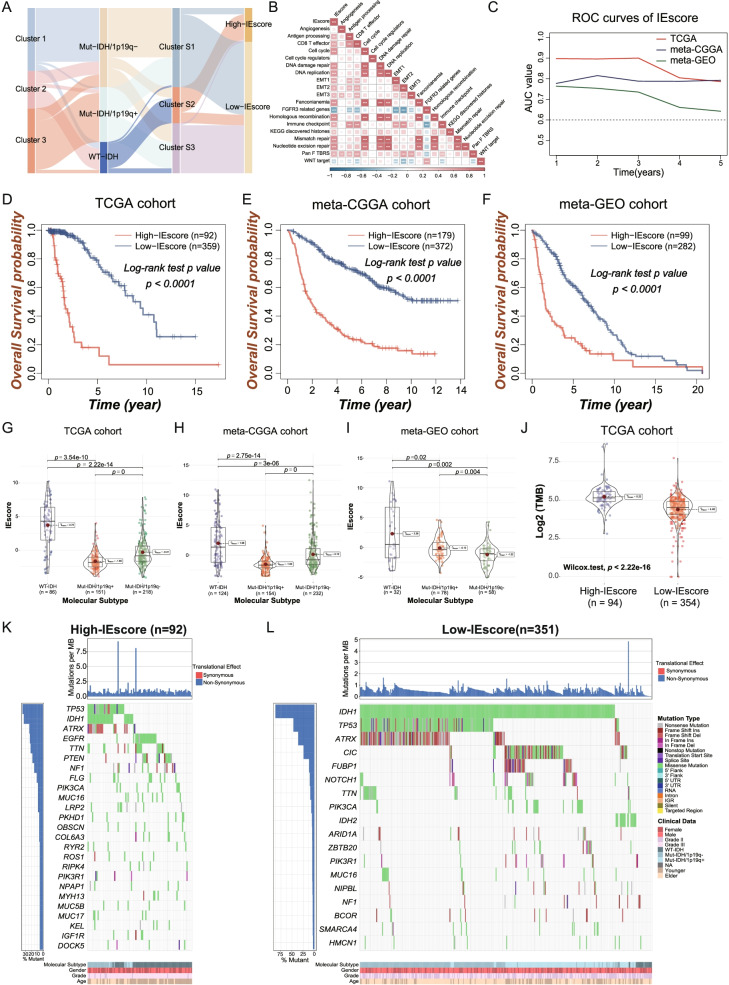
Establishment, prognostic and clinical validation of the IEscore in LGGs. **A** Alluvial diagram of Clusters in group with distinct molecular features, IE-Clusters, and IEscore subgroups. **B** Spearman correlation analysis between IEscore and well-established biological signatures. **C** The ROC curves represented the prognostic predictive of the IEscore in the three LGG cohorts. **D-F** Kaplan–Meier curves indicated that higher IEscore LGG patients showed poorer prognosis compared with lower-IEscore LGG patients in the TCGA (**D**, High-IEscore = 92, Low-IEscore = 359**)**, meta-CGGA (**E,** High-IEscore = 179, Low-IEscore = 372) and met-GEO (**F,** High-IEscore = 99, Low-IEscore = 282**)** LGG cohorts. **G-I** The violin plots indicated that the distinct IEscore levels among the IDH/1p19q molecular subtypes in the TCGA (**G**), meta-CGGA (**H**) and met-GEO (**I**) LGG cohorts. **J** The tumor mutation burden (TMB) level was compared between low-IEscore and high-IEscore LGG subgroups. **K-L** The top frequent mutation genes in high-IEscore LGG patients (**K**, *n* = 92) and low-IEscore LGG patients (**L**, *n* = 351). The labelled asterisk indicated the statistical *p* value (* *p* < 0.05, **p < 0.01 and ***p < 0.001)

Although these findings showed that immune-evasion patterns in LGGs had prognostic implications and were associated with immune cell infiltration, a method of evaluating the immune-evasion level in LGG patients has not yet been determined. IEVscore, a method based on the levels of expression of IE signature genes, was used to quantify the degree of immune evasion in individual patients. The transcriptomic expression profiles of the 607 DEGs were subjected to univariate Cox regression analysis, followed by Lasso regression analysis. These processes identified 17 IESig genes that were used to establish an IEVscore scoring system (Figure S4A, B). The workflow involved in constructing the IEVscore is shown in a Sankey plot (Fig. 6A). To better understand the underlying biological processes and pathways associated with the IEVscore, the statistical correlations between IEVscore and the 19 signatures were determined by Spearman correlation analysis. The resulting correlation heatmap revealed that the IEVscore was positively correlated with the signatures for antigen processing, CD8 T effector and immune checkpoint (Fig. 6B), indicating that higher IEVscores might indicate a higher degree of immune activation in the microenvironment of LGGs.

The ROC curves revealed that IEVscore was a highly robust predictor of OS in LGG patients, with the AUC values of IEVscores predicting 1–5-year OS were > 0.6 in all three patient cohorts (Fig. 6C). Survival analysis indicated that higher IEV scores in all three LGG cohorts were significantly prognostic of poorer outcomes (Fig. 6D–F, p < 0.0001). The associations between IEVscores and the molecular subtypes of LGGs were assessed by comparison analysis, with the results showing that IDH-wild type LGGs were associated with significantly higher IEVscores in all three cohorts, with IDH-mut and 1p/19q co-deletion LGGs having the lowest IEVscores in the TCGA and meta-CGGA cohorts (Fig. 6G-I). Because IEVscore may play a role in response to immunotherapy, the tumor mutation burden (TMB) level was compared in the low- and high-IEVscore subgroups in the TCGA cohort. These comparisons showed that TMB level was significantly higher in the high- than in the low-IEVscore subgroup (Fig. 6J), indicating that IEVscore not only predicts the prognosis of patients with LGG but may also reflect responses to immunotherapy. Furthermore, a waterfall plot comparing the mutational landscapes in the low- and high-IEVscore subgroups in the TCGA-cohort showed the most frequently mutated genes in these subgroups (Fig. 6K, L).

### IEVscore predicts response to immunotherapy

**Fig. 7:**
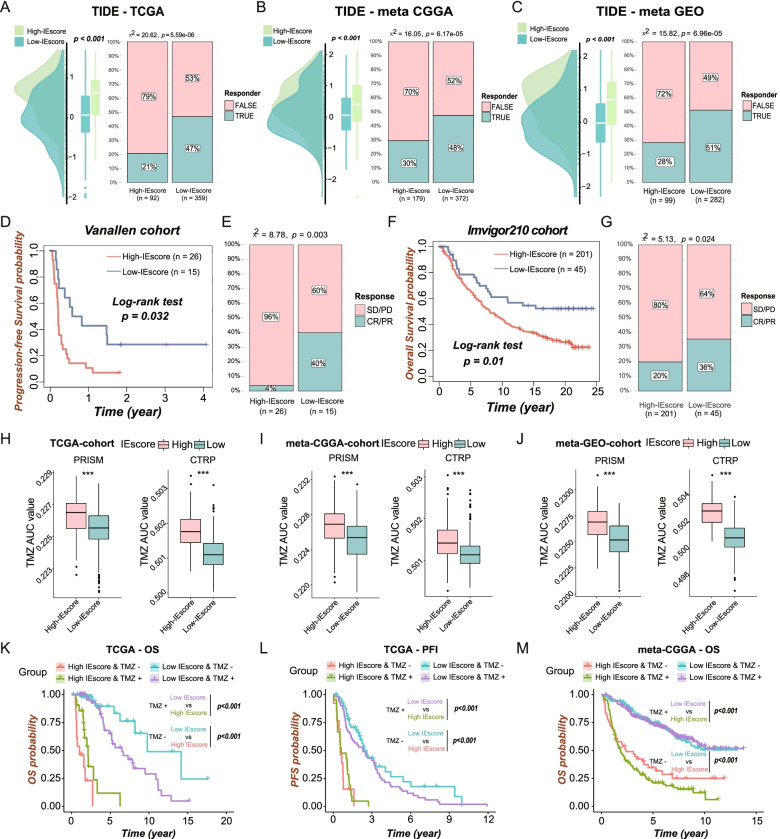
ICI and TMZ sensitivity analysis of IEscore in LGGs. **A-C** The distributions of TIDE scores were compared between low- and high-IEscore groups in the TCGA (**A**), meta-CGGA (**B**) and meta-GEO (**C**) LGG cohorts. **D** Kaplan–Meier curves for low- (*n* = 15) and high-FRscore (*n* = 26) patient groups in Vanallen cohort (melanoma patients treated with anti-CTLA4 therapy) showed higher-IEscore melanoma patients had worse prognosis. **E** Comparison of the response proportions of melanoma patients with anti-CTLA4 therapy between low-(*n* = 15) and high-IEscore (*n* = 26) melanoma patients. **F** Kaplan–Meier curves for low- (*n* = 45) and high-IEscore (*n* = 201) patient groups in IMvigor 210 cohort (cancer patients treated with anti-PD-L1 therapy) showed higher-IEscore cancer patients had worse prognosis. **G** Comparison of the response proportions of cancer patients with anti-PD-L1 therapy between low- and high-IEscore cancer patients. **H-J** The TMZ AUC values, which represented the TMZ resistance level, were calculated using data from PRISM and CTRP datasets respectively, and were compared between low- and high-IEscore LGG subgroup in the TCGA (**H**), meta-CGGA (**I**) and meta-GEO (**J**) LGG cohort. **K-N** Kaplan–Meier curves represent the LGG subgroups of patients with different IEscore levels and TMZ therapy status to reflect the OS (**K**) and PFI (**L**) statuses in the TCGA cohort and OS status in the meta-CGGA cohort **M**. The labelled asterisk indicated the statistical *p* value (***p < 0.001)

To determine whether IEVscore could predict response to cancer immunotherapy, TIDE scores were calculated, and the responses of LGG patients to immunotherapy were predicted using the TIDE algorithm. LGGs with higher IEVscores also showed higher TIDE scores and fewer predicted responders to immunotherapy in all the three LGG cohorts (Fig. 7A–C). Further, IEVscores not only predicted the prognosis of cancer patients treated with immune checkpoint inhibitors (ICIs) but also reflected the immunotherapy response status of cancer patients. Kaplan–Meier survival analysis showed that IEVscore stratified melanoma patients (Vanallen cohort) into two groups with different prognoses, with patients having high-IEVscores showing poorer clinical outcomes (Fig. 7D) and greater resistance to anti-CTLA4 therapy (Fig. 7E). Cancer patients in the IMvigor210 cohort treated with anti-PDL1 could be classified into high- and low-IEVscore groups, with high-IEV score being associated with shorter OS (Fig. 7F) and a lower percentage responding to ICIs (Fig. 7G).

### TMZ sensitivity analysis

Because TMZ is the primary postoperative chemotherapy agent currently used to treat patients with LGG, the correlation between TMZ sensitivity and IEVscore was investigated in these patients. The relative AUC values representing the levels of TMZ resistance were obtained by ridge-regression for the 1421 LGG patients in the Cancer Therapeutics Response Portal (CTRP) and PRISM Repurposing datasets. Patients with high-IEVscore LGG showed greater TMZ resistance in all three LGG cohorts using both the PRISM and CTRP algorithms (Fig. 7H–J). These findings indicated that LGG patients with lower IEVscore might benefit more from TMZ therapy. Subsequent, the prognostic predictive power of IEVscore was investigated in LGG patients in the TCGA and meta-CGGA datasets who were and were not treated with TMZ. Kaplan–Meier survival analysis indicated that IEVscore remained prognostic role and stratified patients who were or were not treated with TMZ into subgroups with distinct prognosis (Fig. 7K–M). Furthermore, Pearson correlation analysis showed that the AUC values, which represent the drug resistance levels of cancer patients, were significantly positively correlated with IEscores in all three LGG cohorts by using the CTRP (Figure S5 A–C) and PRISM (Figure S5 D–F) databases.

## Discussion

The concept of immune evasion has important implications in the tumor microenvironment, malignant progression and drug therapy [44]. Distinct cancer immune evasion patterns were likely derived from intrinsic genomic and transcriptional discrepancies, but the clinical significance of immune evasion status remains unclear [1, 4, 7]. The present study was therefore designed to comprehensively and systematically evaluate the prognostic and therapeutic implications of tumor evasion in LGG. The results of this study may provide clues to the effects of immunotherapeutic strategies in patients with glioma.

This comprehensive investigation showed the genomic alterations and prognostic implications of IEVGs. Unsupervised consensus cluster analysis led to the construction of prognostic signatures based on the IEVGs that were indicative of distinct immune evasion patterns. Of 179 IEVGs analyzed, 93 were significantly upregulated in LGG samples, with the CNV proportions of 130 IEVGs being > 10%. Alterations in IEVGs might be initiating factors of part of LGGs, or immunosuppressive factors which might lead to LGG immune invasion, suggesting the need to understand the functions and mechanisms of these alterations, especially the associations between the IEVGs’ genetic alterations and the formation of immune suppressive microenvironment, and the classical clinicopathological factors of LGGs.

The most important result of the present study was the determination of IEscore and its promising clinical application. The immune evasion related prognostic signature in LGGs was found to be stable, with strong predictive ability to stratify LGG patients by prognosis and response to treatment, in multiple LGG cohorts. Analysis of multi-cohort data showed that higher IEscore was associated with shorter OS, lower TMZ sensitivity and greater resistance to ICI therapy. These results indicated that IEscore is a novel and promising biomarker that can be applied to patients with LGG, identifying patients with a poorer prognosis and guiding their individualized treatment. How to get over the problems in transforming it to clinical applications, by a panel of genes, is the further work we need to finish.

The glioma immune microenvironment differs from the microenvironments of other tumors. Glioma cells can survive in an immune suppressive microenvironment, with intrinsic immune-escape mechanisms playing a major role in the formation and shaping of the immune suppressive microenvironment. Because of the unique features of the glioma microenvironment, the predictive ability of the response of glioma to immunotherapy might not be effective and it should be validated in further glioma ICI cohorts and prospective studies.

Although promising results were found, the present study also had several limitations. First, public transcriptional and clinical data were analyzed retrospectively. Despite the validation of these results in multiple cohorts, prospective studies are needed to validate the identified prognostic signatures. Mechanistic analysis is also necessary to determine the associations of drug sensitivity with IEV-signatures, including the underlying associations between cancer immune escape phenotype and TMZ sensitivity of gliomas. Base on the results we obtained in Fig. 6B, we should realize the potential correlations between IEscore and mismatch repair and nucleotide excision repair pathways. Either the genomic alterations of LGGs result the co-activation of immunological and DNA damage repair pathways, or the interactions between them do? This speculation should be firstly analyzed, and this might explain the discrepancy of TMZ sensitivity between LGGs with distinct IEscore levels.

In conclusion, the present study described a potent prognostic and therapy predictive gene signature for LGG. The IEscore is not only associated with the prognosis, pathway activities and immune cell infiltrations of LGG patients but also reflected sensitivity to TMZ and responses to ICI therapy. IEscore may therefore become a promising postoperative biomarker to predict the clinical outcomes, effects of TMZ chemotherapy and responses to immunotherapy response of patients with LGG.

## Conclusions

In this study, a novel intrinsic immune evasion associated risk signature was established, to predict the clinical prognosis, TMZ sensitivity and potential immunotherapy response of LGG patients. And the IEscore is promising to be used in clinical applications.

## Supplementary Information

**Additional file 1: Figure S1. **The uncropped full-length gels of CEP55 and GAPDH of clinical samples were shown.

**Additional file 2: Figure S2. (A-D) **Unsupervised Consensus Cluster analysis were performed and the cluster heatmaps were visualized when k=2 **(A)**, 3**(B)**, 4**(C)** and 5**(D)**.

**Additional file 3: Figure S3. (A-D) **Unsupervised Consensus Cluster analysis were performed and the cluster heatmaps were visualized when k=2 **(A)**, 3**(B)**, 4**(C)** and 5**(D)**.

**Additional file 4: Figure S4. (A-B) **The Least absolute1 shrinkage and selection operator (LASSO) regression was performed to calculate coefficients **(A) **and the minimum criteria **(B)**.

**Additional file 5: Figure S5.** **(A-F) **The correlation analysis between IEscore and TMZ resistance levels in the TCGA, meta-CGGA and meta-GEO cohorts using CTRP** (A-C) **and PRISM** (D-F) **datasets.

**Additional file 6: Supplementary Table 1.** The list of immune-intrinsic evasion genes.

**Additional file 7. **R codes.

## Data Availability

Totally six original public LGG cohorts including: TCGA-LGG (https://xenabrowser.net/datapages/), CGGA_mRNA_325 and CGGA-mRNA_seq693 (http://www.cgga.org.cn/download.jsp), GSE16011, GSE61374, GSE108474 (https://www.ncbi.nlm.nih.gov/gds) were downloaded from the websites listed. TMZ sensitivity information for 835 cancer cell lines (CCLs) was collected from the Cancer Therapeutics Response Portal (CTRP version.2.0, https://portals.broadinstitute.org/ctrp) database, and 482 CCLs were collected from the Profiling Relative Inhibition Simultaneously in Mixtures (PRISM) Repurposing (https://depmap.org/portal/prism/) database.

## Abbreviations

LGG: Lower-grade glioma
GBM: Glioblastoma
CNS: Central nervous system
GEO: Gene Expression Omnibus
TCGA: The Cancer Genomic Atlas
CGGA: Chinese Glioma Genomic Atlas
RMA: Robust multiarray averaging
ssGSEA: Single sample gene set enrichment analysis
GSVA: Gene Set Variation Analysis
PCA: Principal component analysis
TIDE: Tumor Immune Dysfunction and Exclusion
ICI: Immune check-point inhibitors
TMZ: Temozolomide
CTL: Cytotoxic T lymphocyte
TME: Tumor microenvironment
TMB: Tumor mutation burden
CNV: Copy number variation
SNP: Single nucleotide polymorphisins
CIBERSORT: Cell-type Identification By Estimating Relative Subsets Of RNA Transcripts
DEGs: Differential expression genes
ROC: Receiver operator characteristic
