# Supplementary figures and images for: Intrinsic immune evasion patterns predict temozolomide sensitivity and immunotherapy response in lower-grade gliomas
Source: BMC Cancer. 2022 Sep 12;22:973. doi: 10.1186/s12885-022-09984-5 (PMC9465887; doi:10.1186/s12885-022-09984-5)

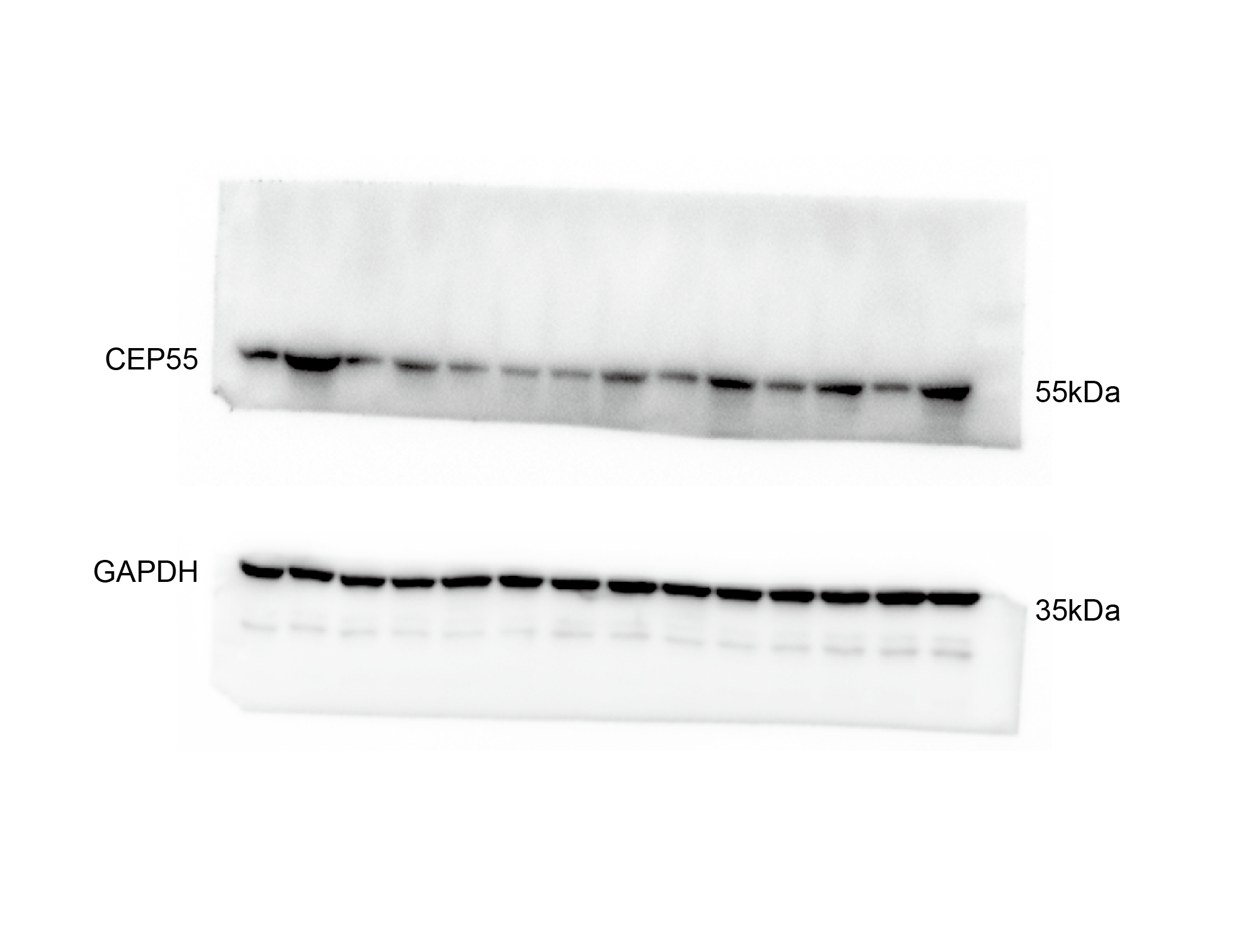

Supplement: Supplementary file 1 — Additional file 1: Figure S1. The uncropped full-length gels of CEP55 and GAPDH of clinical samples were shown. [file 12885_2022_9984_MOESM1_ESM.tif]

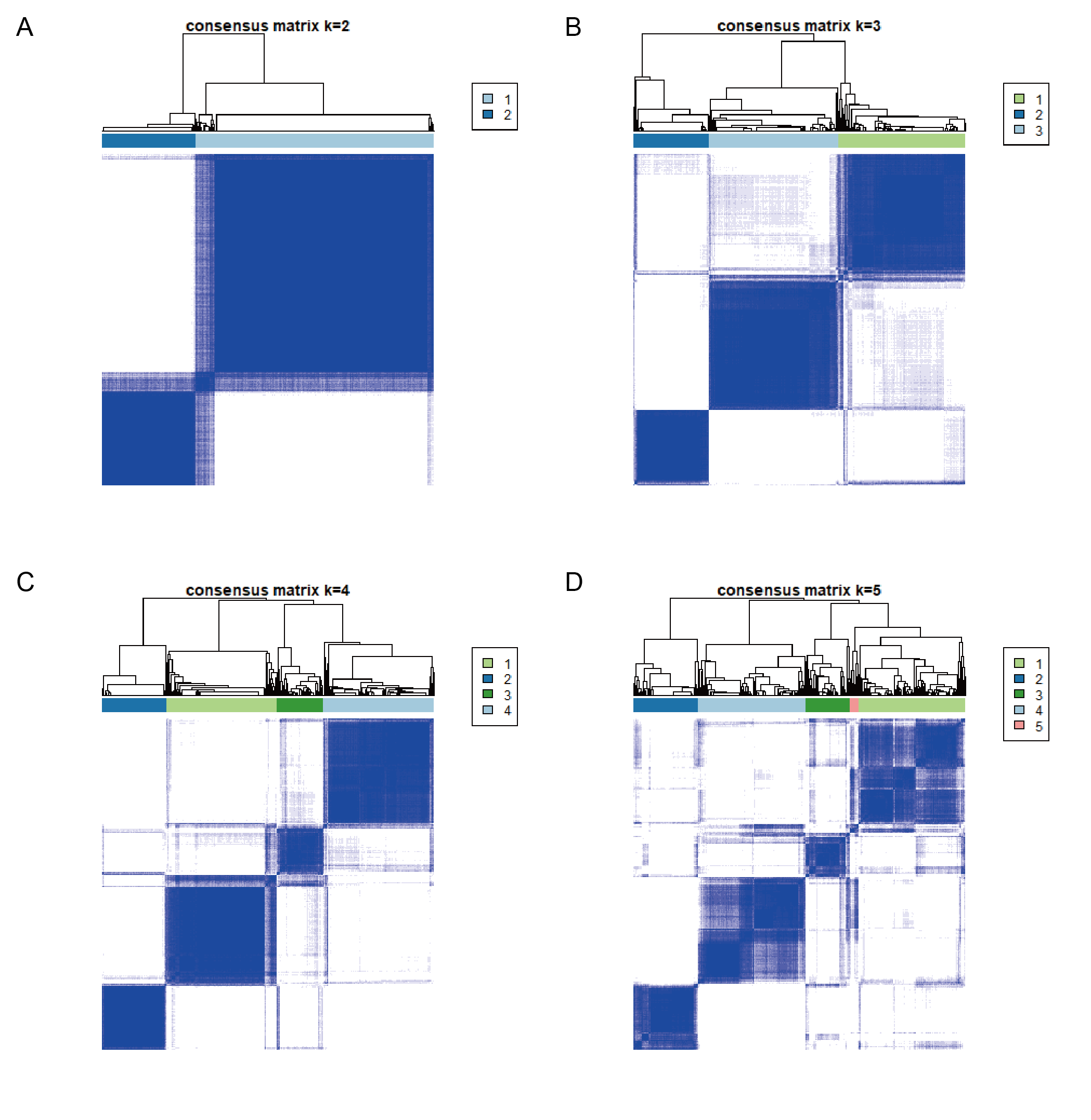

Supplement: Supplementary file 2 — Additional file 2: Figure S2. (A-D) Unsupervised Consensus Cluster analysis were performed and the cluster heatmaps were visualized when k=2 (A), 3(B), 4(C) and 5(D). [file 12885_2022_9984_MOESM2_ESM.tif]

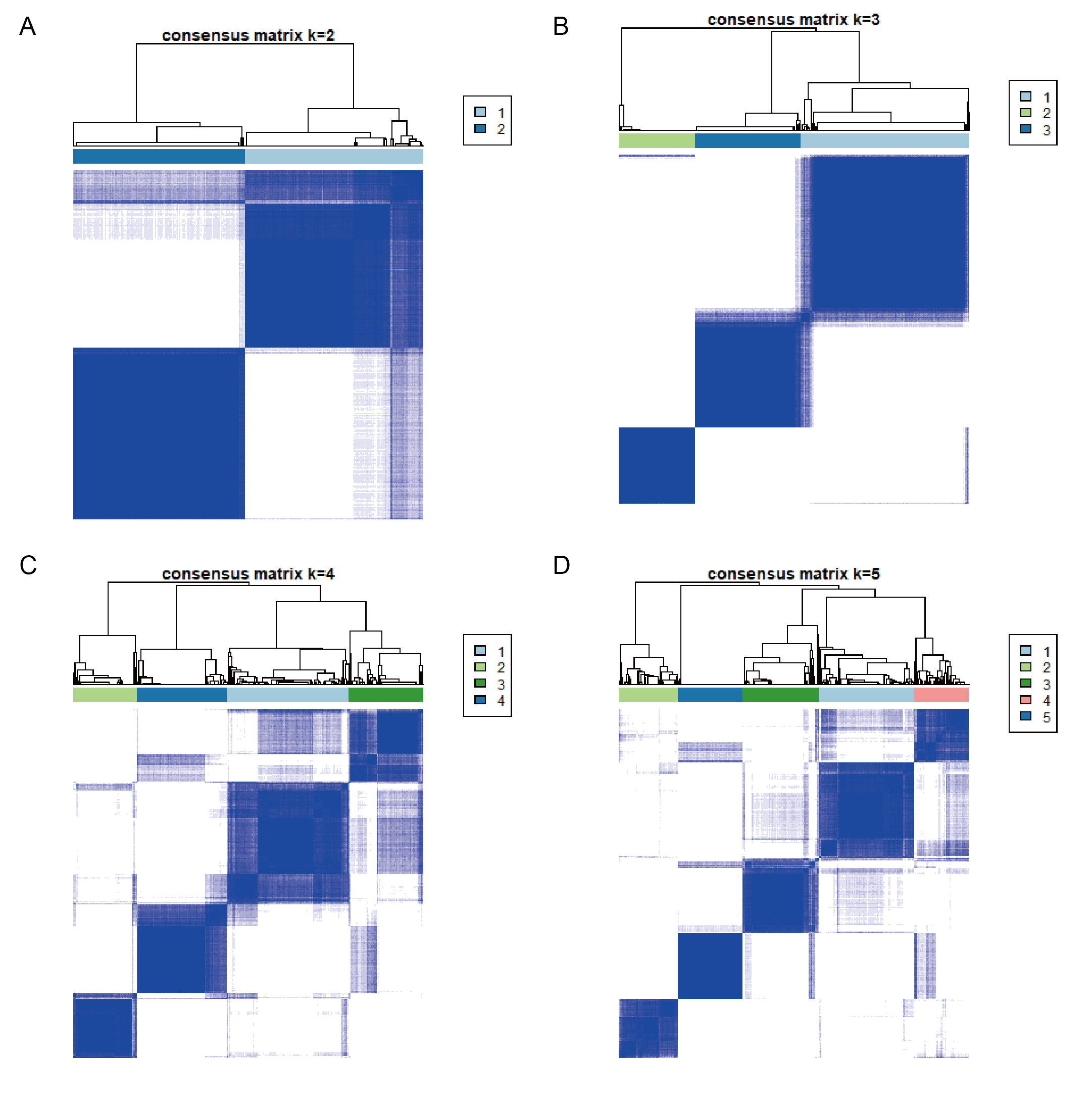

Supplement: Supplementary file 3 — Additional file 3: Figure S3. (A-D) Unsupervised Consensus Cluster analysis were performed and the cluster heatmaps were visualized when k=2 (A), 3(B), 4(C) and 5(D). [file 12885_2022_9984_MOESM3_ESM.tif]

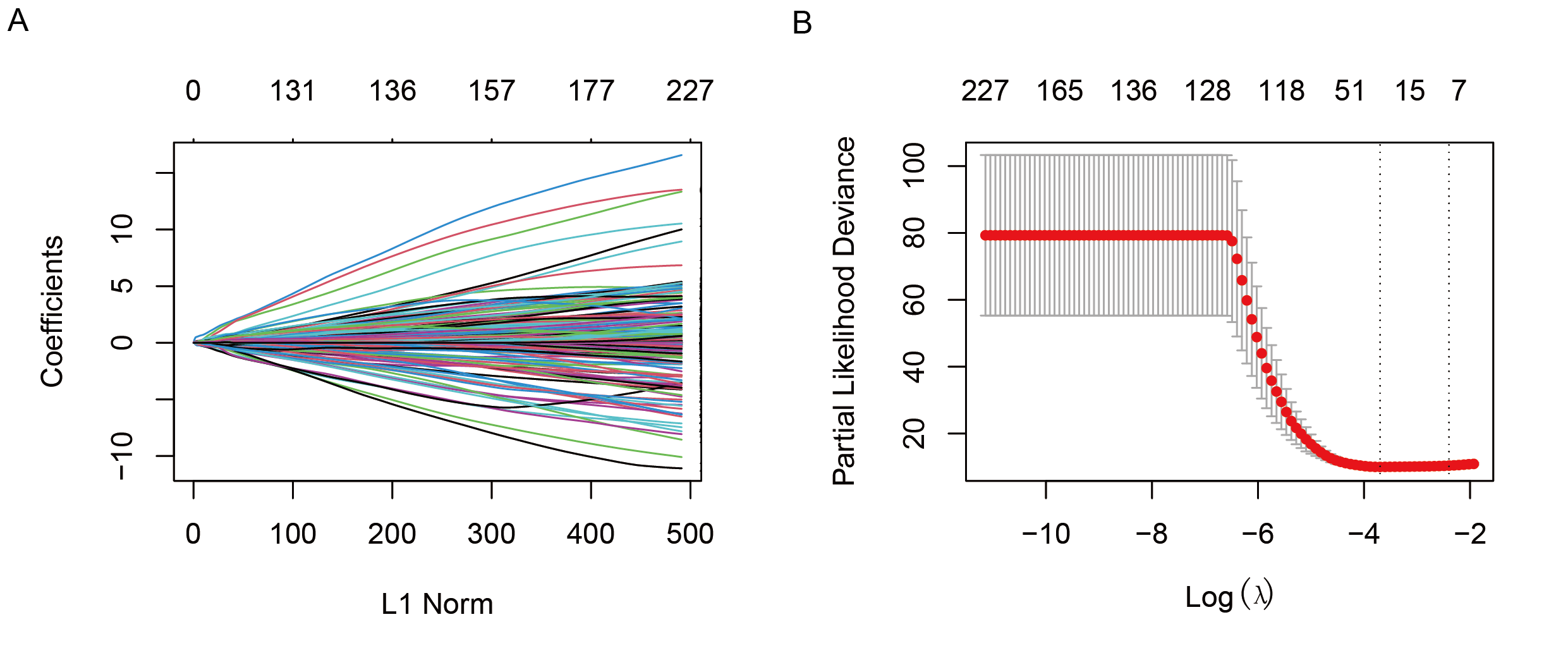

Supplement: Supplementary file 4 — Additional file 4: Figure S4. (A-B) The Least absolute1 shrinkage and selection operator (LASSO) regression was performed to calculate coefficients (A) and the minimum criteria (B). [file 12885_2022_9984_MOESM4_ESM.tif]

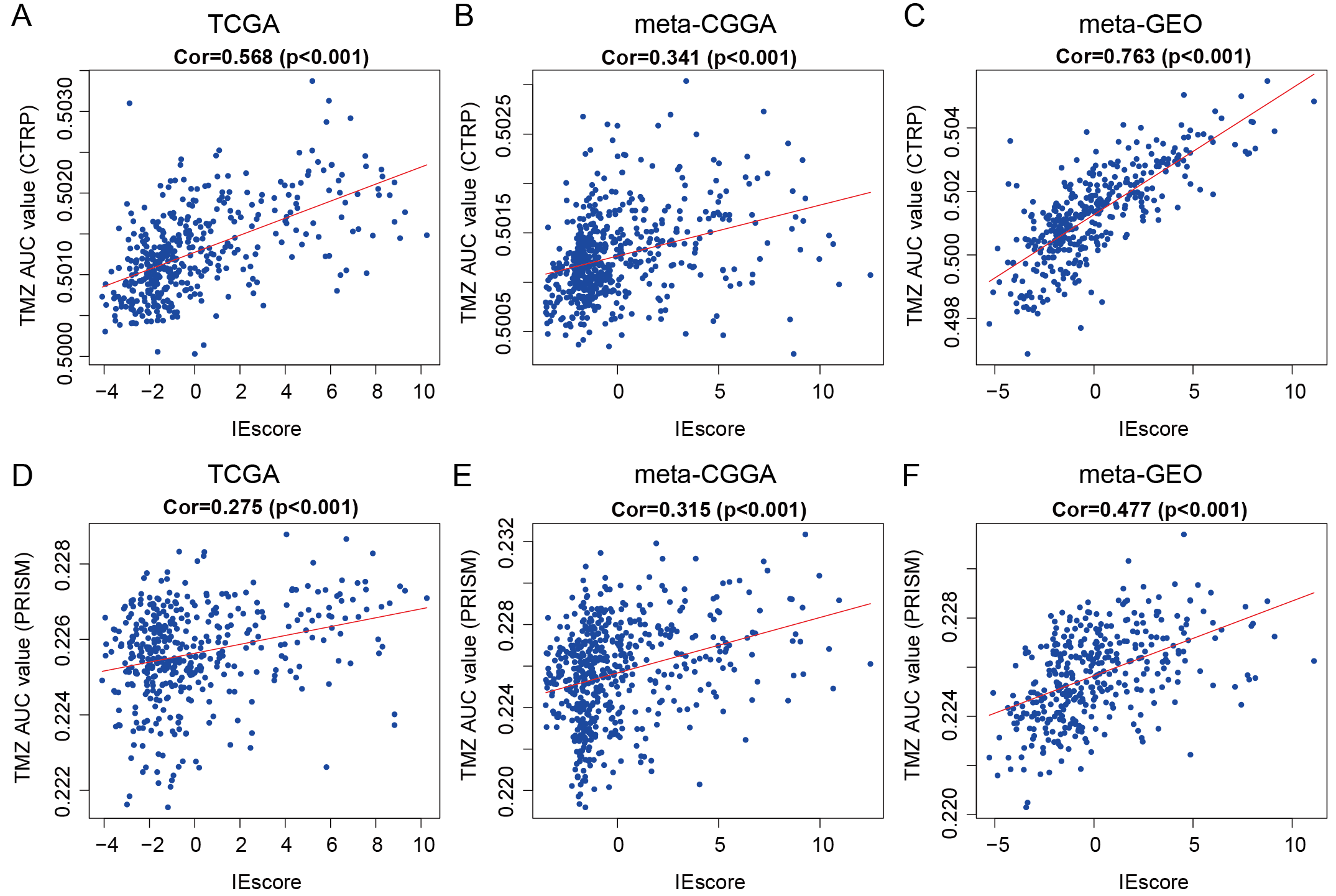

Supplement: Supplementary file 5 — Additional file 5: Figure S5. (A-F) The correlation analysis between IEscore and TMZ resistance levels in the TCGA, meta-CGGA and meta-GEO cohorts using CTRP (A-C) and PRISM (D-F) datasets. [file 12885_2022_9984_MOESM5_ESM.tif]
